# Risk of post-discharge fall-related injuries among adult patients with syncope: A nationwide cohort study

**DOI:** 10.1371/journal.pone.0206936

**Published:** 2018-11-21

**Authors:** Anna-Karin Numé, Nicolas Carlson, Thomas A. Gerds, Ellen Holm, Jannik Pallisgaard, Kathrine B. Søndergaard, Morten L. Hansen, Michael Vinther, Jim Hansen, Gunnar Gislason, Christian Torp-Pedersen, Martin H. Ruwald

**Affiliations:** 1 Department of Cardiology, Copenhagen University Herlev Gentofte Hospital, Hellerup, Denmark; 2 The Danish Heart Foundation, Copenhagen, Denmark; 3 Department of Internal Medicine, Holbæk Hospital, Holbæk, Denmark; 4 Department of Public Health, Section of Biostatistics, University of Copenhagen, Copenhagen, Denmark; 5 Department of Internal Medicine, Nykøbing Falster Hospital, Nykøbing Falster, Denmark; 6 Department of Cardiology, Copenhagen University National Hospital, Copenhagen, Denmark; 7 The National Institute of Public Health, University of Southern Denmark, Copenhagen, Denmark; 8 Departments of Cardiology and Clinical Epidemiology, Aalborg University Hospital, Aalborg, Denmark; University of Malaya, MALAYSIA

## Abstract

**Background:**

Syncope could be related to high risk of falls and injury in adults, but documentation is sparse. We examined the association between syncope and subsequent fall-related injuries in a nationwide cohort.

**Methods:**

By cross-linkage of nationwide registers, all residents ≥18 years with a first-time diagnosis of syncope were identified between 1997–2012. Syncope patients were matched 1:1 with individuals from the general population. The absolute one-year risk of fall-related injuries, defined as fractures and traumatic head injuries requiring hospitalization, was calculated using Aalen-Johansen estimator. Ratios of the absolute one-year risk of fall-related injuries (ARR) were assessed by absolute risk regression analysis.

**Results:**

We identified 125,763 patients with syncope: median age 65 years (interquartile range 46–78). At one year, follow-up was complete for 99.8% where a total of 8394 (6.7%) patients sustained a fall-related injury requiring hospitalization, of which 1606 (19.1%) suffered hip fracture. In the reference group, 4049 (3.2%) persons had a fall-related injury. The one-year ARR of a fall-related injury was 1.79 (95% confidence interval 1.72–1.87, P<0.001) in patients with syncope compared with the reference group; however, increased ARR was not exclusively in older patients. Factors independently associated with increased ARR of fall-related injuries in the syncope population were: injury in past 12 months, 2.39 (2.26–2.53, P<0.001), injury in relation to the syncope episode, 1.62 (1.49–1.77, P<0.001), and depression, 1.37 (1.30–1.45, P<0.001)

**Conclusion:**

Patients with syncope were at 80% increased risk of severe fall-related injuries within the year following discharge. Notably, increased risk was not exclusively in older patients.

## Introduction

Syncope episodes are frequent in both young and older adults, [1–3] and characterized by a total loss of consciousness due to transiently reduced cerebral blood flow with subsequent complete recovery. [4, 5] Nonetheless, episodes do often lead to falls, and syncope could be related to an increased risk of injuries. Falls and fall-related complications are a considerable public health concern in terms of morbidity, mortality, quality of life, and cost of health and social services, especially among older adults. [6–10]

Over the last decade, the overlap in symptoms of syncope and falls, particularly in older persons, has received growing attention. [11–13] A number of age-related factors (physiological and pathological), in combination with amnesia for loss of consciousness, and lack of a witness account, may confound the assessment of syncope. [14] Consequently, persons with syncope are likely to present with an unexplained fall rather than syncope. [15, 16] Moreover, several studies have observed high prevalence of cardiovascular conditions among older persons presenting with unexplained falls. [17] Some of these conditions, particularly carotid sinus syndrome and orthostatic hypotension, are observed risk factors for unexplained falls and fall injuries, and also important causes of syncope in the elderly. [18–21] Yet, evidence on the associations between syncope syndromes and falls or injuries is sparse and mainly based on cross-sectional studies in selected settings. One study among approximately 200 elderly patients with syncope reported a two-year incidence of fractures of 11%, but injury was a secondary outcome and further analysis was not undertaken. [22] Moreover, previous studies among adult patients with syncope report that 26% to 39% suffer from injuries in relation to their syncope episode, [23, 24] but whether this is exclusively in older adults is unknown.

We conducted a nationwide study of adult patients with a first-time diagnosis of syncope, to provide longitudinal population-based data on the association between syncope and subsequent fall-related injuries. Our objectives were to assess the risk of fall-related injuries following syncope and evaluate if physical injury in this population occurs predominantly in older adults, and to compare the risk of fall-related injuries following syncope with that of the general population.

## Methods

The study is a nationwide register-based cohort study from January 1, 1997 to December 31, 2013. The study was conducted in Denmark where health services are predominantly tax-funded, which ensures free access to healthcare for the entire population. Individual-level linkage of information between population-based registers is possible due to a unique and personal identification number, which is assigned to each resident at birth or upon immigration. [25]

### Registers

The Civil Registration System holds information on the date of birth, death, sex, and migration for all residents. [25] Data on medical history and outcomes were retrieved from discharge diagnoses and claimed prescriptions as appropriate. The Danish National Patient Register holds data on all hospitalizations since 1977. [26] At discharge, each hospital contact is registered with one principal diagnosis, and if appropriate, one or several supplementary diagnoses according to the International Classification of Diseases (ICD). The Danish Register of Medicinal Product Statistics comprises information about claimed prescriptions, and each drug dispensing is registered according to the Anatomical Therapeutic Chemical classification system. [27] Partial reimbursement of drug expenses by the national healthcare system ensures complete registration by the pharmacies. Average five-year household income prior to study start served as a proxy of socioeconomic status, and information was obtained from Statistics Denmark. [28]

### Study population

The study population comprised all residents ≥18 years with a first-time primary discharge diagnosis of syncope between 1997 and 2012. Both inpatient and emergency department (ED) encounters were included. Subjects with prior syncope outpatient contacts were excluded. The reference group was obtained with risk set matching: Each subject in the syncope population was matched with one subject from the general population without prior syncope hospitalizations by year of birth and sex. The ICD diagnosis of syncope (10th revision code R55.9) refers to the most common etiologies of syncope, [5] and has previously been validated with a positive predictive value of 96%.[29]

### Covariates

Potential confounding factors were pre-specified and identification was based on current knowledge in combination with the construction of a model diagram. [30] The following medical variables were considered: cardiovascular disease (including ischemic heart disease or myocardial infarction, heart failure, cardiac arrhythmia, atrioventricular block or left bundle branch block, cerebral vascular disease, or peripheral vascular disease), pacemaker, diabetes mellitus, cancer, dementia, depression, Parkinson disease, and use of loop diuretic, antihypertensive, or anxiolytic drugs. Information was retrieved from diagnosis or surgical procedure codes up to ten years prior to inclusion, and from claimed prescriptions up to one year prior to inclusion (S1 Table). We considered combination treatment with at least two standard antihypertensive agents within a period of 90 days as use of antihypertensive drugs. [31] When appropriate, we combined diagnosis and prescription data of comorbidities such as diabetes mellitus, depression, and dementia, to increase the sensitivity of the covariates. Osteoporosis is a main risk factor of fragility-fractures, but because the association with syncope is unclear, it was not considered a principal confounder.

### Study outcome

The primary outcome of a fall-related injury was defined as any hospital encounter (ED visit or inpatient admission) from fractures (femur, pelvis, shoulder or upper arm, elbow, forearm or wrist, and skull) or traumatic head injuries (S1 Table). The approach has been used previously as a proxy for serious falls. [9] The outcome of interest was post-discharge injuries, so documentation of an injury in relation to the syncope episode was not considered as an event (but evaluated in supplementary analyses). The study population was followed for one year, or until the occurrence of a fall-related injury, emigration, or death.

### Statistics

Differences in baseline characteristics were compared using chi-squared test for categorical variables. We report loss to follow-up at one year. The level of significance was set at 5%. To assess the time-dependent absolute risk of fall-related injuries following syncope, we used the Aalen-Johansen estimator to account for the competing risk of death. [32] Furthermore, we report one-year risks separately for the syncope and reference group and according to age at baseline. The relation between the absolute risks and age (continuous scale) was obtained with the Aalen-Johansen estimate and kernel smoothing.

In our main analysis, we performed absolute risk regression analysis, [33] and report absolute risk ratios (ARR) with 95% confidence intervals (CI) referring to the probability of sustaining a fall-related injury during the next year for persons with syncope compared to persons without syncope, given fixed values for the other predictor variables. Models were adjusted for age (five-year intervals), sex, calendar year (four-year intervals), and socioeconomic status in addition to comorbidities and pharmacotherapies. Effect modification was analyzed in subgroups defined by clinical relevance, thus the syncope-associated one-year risks of fall-related injuries were estimated in subgroups defined by age, sex, cardiovascular disease, arrhythmia, pacemaker, loop diuretic use, depression, and fall-related injury in the past 12 months. We further analyzed absolute risk ratios to associate changes in one-year risk of fall-related injuries with differences in person characteristics in the syncope population.

Sensitivity analyses adjusted for osteoporosis and prior fall-related injury respectively, and in another analysis, we excluded all patients with fall-related injury in relation to the syncope episode. We also examined whether the risk was similar for patients with syncope who were admitted to hospital and patients discharged from the ED. All analyses were repeated with hip fracture as the outcome of interest; specifically, because hip fractures are both highly correlated with falls, and require hospitalization. Statistical analyses were performed using SAS version 9.4 (SAS Institute Inc., Gary, NC, USA) and R version 3.4. [34]

#### Ethics

The study was approved by the Danish Data Protection Agency (ref. number: 2007-58-0015 / GEH-2014-013 I-Suite number: 02731). In Denmark, ethical approval is not required for retrospective register-based studies. All analyses were executed on servers placed at Statistics Denmark.

## Results

In the period from 1997 through 2012, 125,763 adult patients with a first-time diagnosis for syncope were identified (n = 8288 were excluded due to prior syncope outpatient contacts), of which 68,671 (54.6%) represented inpatient admissions. The median age of patients with syncope was 65 years (interquartile range [IQR] 46–78) and 65,608 (52.2%) were women (Table 1). The most prevalent comorbidities were ischemic heart disease (n = 20,093, 16.0%), arrhythmia (n = 15,673, 12.5%), and depression (n = 22,415, 17.8%). Prevalence of comorbidities was greater in the syncope population compared with the matched reference group, as was the frequency of a prior fall-related injury (n = 7912, 6.3% versus n = 3774, 3.0%, P<0.001).

**Table 1:** Baseline characteristics of the study population^a^^,^ ^b^.

	Syncope (n = 125,763)	No syncope (n = 125,763)
Age, median [IQR], years	65 [46–78]	65 [46–78]
Age groups, years		
18–49	35,213 (28.0)	35,213 (28.0)
50–64	26,292 (20.9)	26,292 (20.9)
65–79	36,153 (28.7)	36,153 (28.7)
≥80	28,105 (22.3)	28,105 (22.3)
Women	65,608 (52.2)	65,608 (52.2)
Men	60,155 (47.8)	60,155 (47.8)
Income group^c^, quartiles		
<First quartile	24,933 (19.8)	25,372 (20.2)
First to third quartile	78,049 (62.1)	72,867 (57.9)
>Third quartile	22,781 (18.1)	27,524 (21.9)
Comorbidity		
Cardiovascular disease	40,659 (32.3)	20,031 (15.9)
Ischemic heart disease or MI	20,093 (16.0)	9428 (7.5)
Heart failure	9053 (7.2)	4033 (3.2)
Cardiac arrhythmia	15,673 (12.5)	6679 (5.3)
Atrial fibrillation	11,688 (9.3)	5339 (4.2)
AV block or LBBB	2576 (2.0)	691 (0.5)
Cerebral vascular disease	12,875 (10.2)	5755 (4.6)
Pacemaker	3700 (2.9)	937 (0.7)
Diabetes mellitus	10,500 (8.3)	7123 (5.7)
Cancer	10,378 (8.3)	7953 (6.3)
Depression	22,415 (17.8)	12,806 (10.2)
Parkinson disease	2760 (2.2)	1499 (1.2)
Dementia	3981 (3.2)	2097 (1.7)
Osteoporosis	5892 (4.7)	4779 (3.8)
Pharmacotherapy		
Antihypertensive drugs	37,865 (30.1)	25,480 (20.3)
Loop diuretic drugs	17,910 (14.2)	11,330 (9.0)
Anxiolytic drugs	27,041 (21.5)	18,332 (14.6)
Fall-injury in past 12 m	7912 (6.3)	3774 (3.0)
Syncope inpatient admission	68,671 (54.6)	NA
Syncope ED visit	57,092 (45.4)	NA
Syncope and injury^d^	4601 (3.7)	NA
Year of inclusion		
1997–2000	29,776 (23.7)	29,776 (23.7)
2001–2004	32,640 (26.0)	32,640 (26.0)
2005–2008	31,171 (24.8)	31,171 (24.8)
2009–2012	32,176 (25.6)	32,176 (25.6)

Abbreviations: IQR (interquartile range), MI (myocardial infarction), LBBB (left bundle branch block), ED (emergency department), NA (not applicable)

^a^Data are expressed as no. (%) unless otherwise indicated

^b^P values were <0.001 (except for age and sex)

^c^Average five-year household income prior to inclusion

^d^Documented fall-related injury in relation to the syncope episode

### Absolute one-year risk of fall-related injuries following syncope

**Fig 1:**
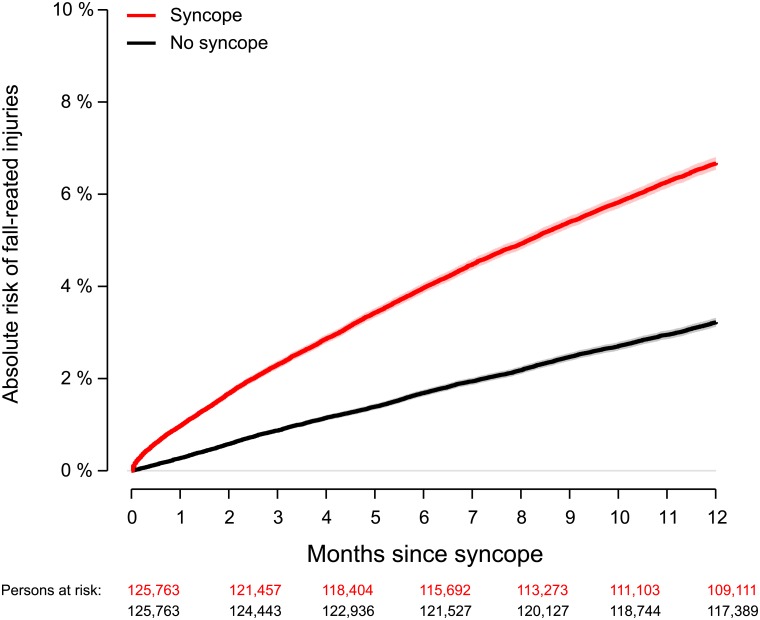
Absolute risk of hospitalization due to fall-related injuries following syncope. Syncope population (red), matched reference group (black). One-year absolute risk of fall-related injuries was 6.7% (95% CI, 6.5%-6.8%) in the syncope population, and 3.2% (95% CI, 3.1%-3.3%) in the age- and sex matched reference group.

**Fig 2:**
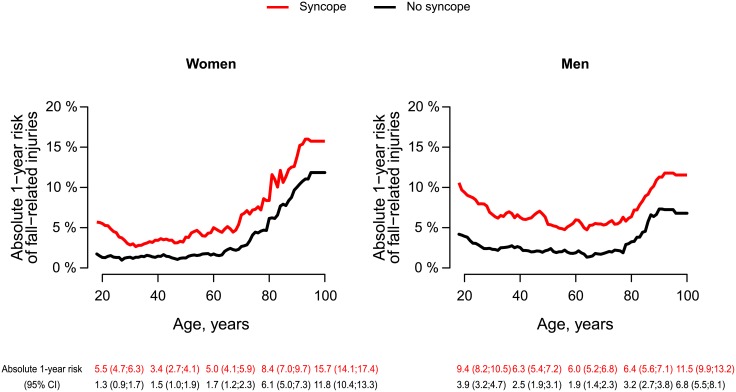
Absolute one-year risks of fall-related injuries according to age. Absolute one-year risks of fall-related injuries according to age at syncope accounting for the competing risk of death in the syncope (red) and matched reference population (black) respectively. Selected point estimates with 95% CI are shown.

At one year, follow-up was complete for 99.8% of the syncope population (n = 199, 0.2% emigrated). A total of 8394 (6.7%, 95% CI, 6.6%-6.8%) patients had a fall-related injury requiring re-hospitalization, whereas 4049 (3.2%, 95% CI, 3.1%-3.3%) persons in the reference group had a fall-related injury (Fig 1). Hip fracture accounted for one out of five injury hospitalizations among persons with syncope (n = 1606, 19.1%) compared with n = 1016 in the reference group (S2 Table). Fig 2 shows the absolute one-year risks of fall-related injuries according to age in the syncope and reference population respectively. The absolute risk of a fall-related injury increased with advancing age, and was particularly high among elderly women; however, young men did also have a substantial risk of injury.

### Risk of fall-related injuries in patients with syncope compared to persons without syncope

**Fig 3:**
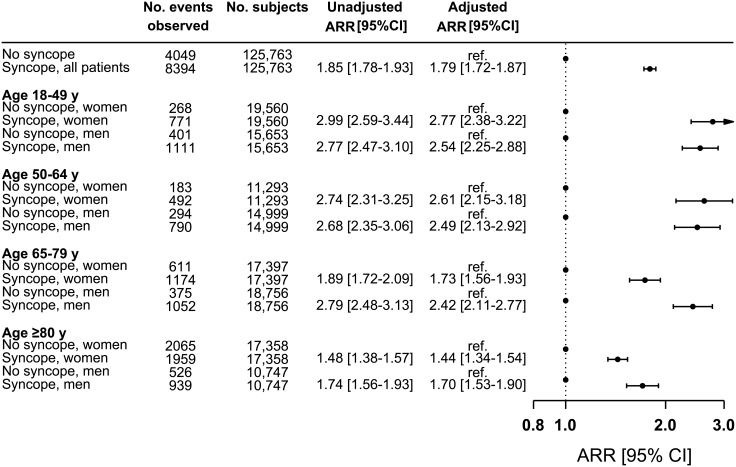
One-year absolute risk ratios of fall-related injuries in the syncope population compared with the reference. The matched group without prior syncope served as reference within each stratum. Multiple absolute risk regression analyses with adjustment for: age, sex, socioeconomic status, calendar year, ischemic heart disease, arrhythmia, atrioventricular block or left bundle branch block, pacemaker, use of antihypertensive, loop diuretic or anxiolytic drugs, depression, diabetes, cancer, Parkinson disease, and dementia. Number of events refers to observed events within one year. ARR indicates absolute risk ratio.

The one-year adjusted ARR of fall-related injuries was 1.79 (95% CI, 1.72–1.87, P<0.001) in patients with syncope compared with the reference group (unadjusted ARR, 1.85, 95% CI, 1.78–1.93, P<0.001). Fig 3 presents risk estimates stratified by age and sex. We found that the ARR decreased with advancing age. Also, in the older age groups, the relative importance of syncope was greater in men (65–79 years: ARR, 2.42, 95% CI, 2.11–2.77) compared with women (65–79 years: ARR, 1.73, 95% CI, 1.56–1.93). S1 Fig provides a summary of subgroup analyses. The syncope-associated one-year risks of fall-related injuries were increased across subgroups, with the exception of patients with pacemaker (ARR, 1.19, 95% CI, 0.89–1.59, P = 0.23) versus no pacemaker (ARR, 1.81, 95% CI, 1.73–1.88, P<0.001).

### Characteristics associated with one-year risk of fall-related injuries

**Fig 4:**
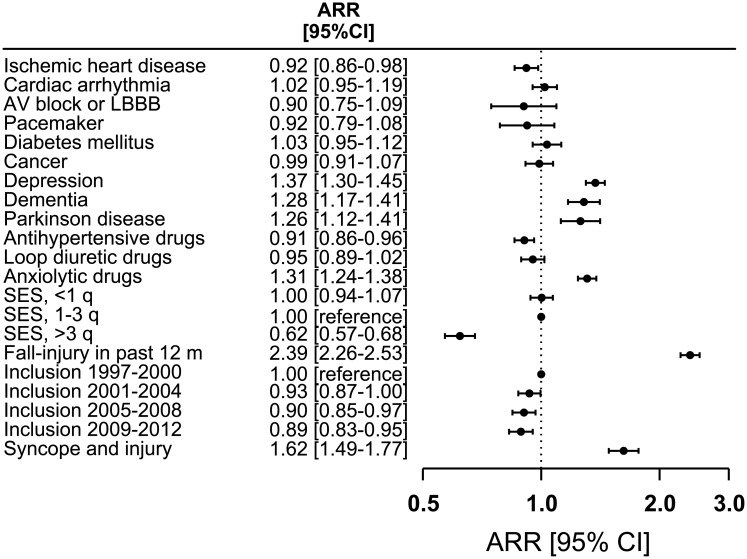
Person characteristics associated with one-year absolute risk ratios of fall-related injuries in the syncope population. Multiple absolute risk regression analysis was done to associate changes in one-year risks of subsequent fall-related injuries with person characteristics. ARR indicates absolute risk ratio, LBBB left bundle branch block, and SES socioeconomic status. Syncope and injury refers to a documented injury in relation to the syncopal event.

The three factors most associated with increased ARR of fall-related injuries in the syncope population were: fall-related injury in past 12 months (2.39, 95% CI, 2.26–2.53, P<0.001), fall-related injury in relation to the syncope episode (1.62, 95% CI, 1.49–1.77, P<0.001), and depression (1.37, 95% CI, 1.30–1.45, P<0.001), whereas high socioeconomic status, and use of antihypertensive drugs were associated with decreased one-year risk of fall-related injuries (Fig 4).

### Sensitivity analyses

The syncope-associated one-year risk of fall-related injuries was similar for patients seen in the ED (ARR, 1.84, 95% CI, 1.72–1.96, P<0.001) and patients admitted to hospital (ARR, 1.77, 95% CI, 1.68–1.87, P<0.001). Adjustment for osteoporosis did not challenge the results from the main analysis (ARR, 1.79, 95% CI, 1.72–1.86, P<0.001), neither did adjustment for prior fall-related injury (ARR, 1.72, 95% CI, 1.65–1.80, P<0.001), or exclusion of patients with fall-related injury in relation to their syncope episode (ARR, 1.74, 95% CI, 1.67–1.81, P<0.001). Moreover, the main analysis was repeated with hip fracture as outcome, but yielded similar results among subjects’ ≥65 years (the analysis could not be executed in younger subjects due to a limited number of events), S2 Fig.

## Discussion

The main finding of the study was that first-time syncope was associated with 80% increased one-year risk of fall-related injuries, in terms of fractures and traumatic head injuries requiring hospitalization, compared with an age- and sex matched reference group. The study is the first to provide comprehensive longitudinal data on injury risk in a real-world clinical setting of adult patients with syncope. Although the absolute risk increased with advancing age, the relative risk of fall-related injuries associated with syncope decreased in the elderly. Plausibly, because multiple factors influence fall-injury risk in older persons the attributable risk associated with syncope decreases with advancing age. Notably, increased risk of injury was not exclusively in older patients, but also substantially increased in young adults with syncope. Depression, prior fall-related injury, and injury in relation to the syncope episode were important risk factors associated with increased risk of sustaining a fall-related injury.

Our observation of increasing absolute risk of fall-related injuries with advancing age, particularly in older women is in line with current knowledge. [9, 35, 36] Medical conditions and physiological changes associated with aging such as impairment in cognition and declines in balance, gait, and muscle strength, contribute to an increased likelihood of falls amongst older adults. [36, 37] In addition, bone fragility such as osteoporosis increases susceptibility to serious injury. [38] Both sarcopenia (age-associated loss of skeletal muscle mass and function) and bone fragility accelerates in association with menopause-related estrogen-deficiency, [39] which could explain the difference in relative risk observed between men and women with syncope in the older age groups.

Although the majority of injuries were observed in the elderly population, younger patients with a history of syncope also had markedly increased risk of injury. However, the injury mechanism is liable to be different in younger and older adults, and while the injuries identified in the current study are strongly related to falls in older adults, [40] other causes may predominate in younger adults such as transportation, work, and sport or leisure activities.[35] Information pertaining to the specific circumstances of the sustained injuries was unavailable in the current study.

Our findings are in keeping with prior research on the association of some individual causes of syncope and unexplained falls and fall injuries in older persons.[18,19,21,41] One study observed that amongst older persons with dementia, roughly 50% of individuals initially referred for an unexplained fall was eventually diagnosed with syncope, with orthostatic hypotension as the most common cause.[42] Similarly, it has been reported that prevalence of CSS is common in older persons referred to ambulatory management for unexplained falls, ranging from 14% to 27%.[20,41,43] Comparisons of our risk estimates regarding the effect of syncope with previous studies remain difficult due to substantial differences in study design, setting, and demographics of the study population.

With advancing age, the attributable risk of cardiac causes of syncope increases. We observed that in patients with a cardiac pacemaker, syncope was not significantly associated with risk of subsequent fall-related injuries. This is in line with a study, which demonstrated a substantial reduction in the number of injurious events amongst individuals with cardio-inhibitory carotid sinus hypersensitivity randomized to pacing.[44] However, conflicting results have been observed for effects of pacing intervention on fall-rates.[45] Furthermore, atrial fibrillation has been associated with syncope and falls.[46, 47] In one study among older adults presenting with unexplained falls, 71% had an incident cardiac arrhythmia within less than a year using an implantable loop recorder, and in 20% a recurrent fall and/or syncope was directly attributable to the arrhythmia. [48] In contrast with these observations, we demonstrated that the syncope-associated risk of fall-related injuries was relatively lower among persons with pre-existing arrhythmias and cardiovascular disease in general. However, register-based data could be subject to ascertainment bias i.e. the probability of receiving a specific cardiac diagnosis over a non-specific syncope diagnosis could be increased in more grievous cases.

Although a single cause is evident in a minority of falls, a multitude of factors, such as environmental factors, acute and chronic medical conditions, and medications are likely to influence the risk of falling. [36, 49] Medications with orthostatic effects, such as antihypertensive, diuretic, and psychotropic drugs, have repeatedly been associated with both syncope and falls. [50–52] In contrast, we observed that use of antihypertensive and loop diuretic drugs was not associated with increased risk in patients with syncope; however, dose changes were not accounted for and could be a possible explanation. Moreover, the additive effects of multiple present risk factors were out of the scope of the current study. In one study, [37] the probability of (recurrent) falls increased from 3% amongst persons with no documented risk factors to 84% among persons with five or more risk factors indicating that modifying even a few factors may reduce the risk of fall-related injuries. Cardiovascular assessment is fundamental in the evaluation of patients with syncope, but perhaps a more extensive assessment is required in some patients. Our results demonstrating that depression, use of anxiolytic drugs, dementia, and prior injury were associated with subsequent re-hospitalization due to fall-related injuries could plausibly indicate that a more multifactorial and interdisciplinary approach needs to be considered in the elderly and patients with multimorbidity.[4, 53, 54] Establishment of formal syncope management facilities could be one way to ensure standardized and appropriate management of patients with syncope, despite the heterogeneous patient group composition, but further research is required. [55]

### Strengths and limitations

The main strength of this study was the use of nationwide registers that enabled identification and complete follow-up of a large cohort of patients with syncope irrespective of age, morbidity, socioeconomic status, and health insurance schemes, and with independent data collection of outcomes. However, the study has several potential limitations. First, due to the observational design, direct conclusions on causal relationships of our findings remain unviable. Second, important clinical parameters including blood pressure, electrocardiographic, echocardiographic or carotid sinus massage findings were unavailable in the registries, so despite extensive adjustments for potential confounding factors, we cannot exclude effects of residual confounding. Furthermore, the ICD registration does not specify the etiologies of syncope; consequently, we do not address effect measures attributable to specific causes of syncope. Instead, we have evaluated subgroups, mainly within different cardiovascular conditions. Third, we cannot exclude the possibility that persons who contact the ED or hospital due to syncope may be inherently different from those who do not; however, effect measures of hip fracture, which should always lead to hospitalization, supported the main results. Of note, the reported risk of fall-related injuries only reflects the number of injury events coming to acute medical attention at EDs and hospitals, and may, therefore, represent an underestimate of total fall-related events. Information pertaining to fatal fall incidents resulting in immediate death prior to hospitalization was unavailable.

### Conclusions

In this nationwide study, adult patients with first-time syncope were at 80% increased risk of severe fall-related injuries within the year following discharge. Notably, increased risk was not exclusively in older patients. These findings underscore the necessity of increased clinical awareness about traumatic injury risk and appropriate management of patients with syncope of all ages in clinical practice, and support increased multidisciplinary assessment in falls-prevention among older adults. Further cohort and intervention studies will be needed to reveal causal effects and efficacy of preventive strategies.

## Supporting information

S1 Table Specific codes used to describe the study population and to identify endpoints. (PDF)

S2 Table Summary of fall-related injures within one year. (PDF)

S1 Fig Subgroup analyses of the one-year absolute risk ratios of fall-related injuries between syncope population and reference. The age- and sex matched group without prior syncope served as reference in all analyses. Multiple absolute risk regression analyses with adjustment for: age, sex, calendar year, socioeconomic status, comorbidities, and pharmacotherapy. ARR indicates absolute risk ratio.
(PDF)

S2 Fig One-year absolute risk ratio of hip fractures in the syncope population ≥65 years compared with the reference. The age- and sex matched group ≥65 years served as reference. Multiple absolute risk regression analyses with adjustment for: age, sex, calendar year, socioeconomic status, ischemic heart disease, arrhythmia, atrioventricular block or left bundle branch block, pacemaker, use of antihypertensive, loop diuretic or anxiolytic drugs, depression, diabetes, cancer, Parkinson disease, and dementia. ARR for total fall-related injury is provided for comparative purpose. ARR indicates absolute risk ratio.
(PDF)
