# Supplementary material for: Risk of post-discharge fall-related injuries among adult patients with syncope: A nationwide cohort study
Source: PLoS One. 2018 Nov 21;13(11):e0206936. doi: 10.1371/journal.pone.0206936 (PMC6248940; doi:10.1371/journal.pone.0206936)
Supplement: S1 Table — (PDF) [file pone.0206936.s001.pdf]

S1 Table. Specific codes used to describe the study population and to identify endpoints

	Defined from	Codes used
Syncope	Diagnosis codes	(ICD8: 7802) ICD10: R55.9
**Comorbidity and pharmacotherapy**		
Ischemic heart disease including MI	Diagnosis codes	ICD10: I20-25
Cerebral vascular disease	Diagnosis codes	ICD10: I60-69
Heart failure	Diagnosis codes	ICD10: I110, I130, I132, I42, I50
Peripheral vascular disease	Diagnosis codes	ICD10: I70, I74
AV block and LBBB	Diagnosis codes	ICD10: I44
Arrhythmia	Diagnosis codes	ICD10: I47-49
Aortic stenosis	Diagnosis codes	ICD10: I350, I352
Diabetes mellitus	Diagnosis codes or prescriptions	ICD10: E10-E14; ATC: A10
Cardiac pacemaker	Surgical procedure codes	NCSP: BFCA0-2, KFPE0-2, KFPF0-2
Depression	Diagnosis codes or prescriptions	ICD10: F32-33; ATC: N06A
Cancer	Diagnosis codes	ICD10: C00-C96
Dementia	Diagnosis codes or prescriptions	ICD10: G30; ATC: N06D
Parkinson disease	Diagnosis codes or prescriptions	ICD10: G20; ATC: N04
Osteoporosis	Diagnosis codes or prescriptions	ICD10: M80-M85; ATC: M05B
Loop diuretic drugs	Prescriptions	ATC: C03CA
Antihypertensive drugs	Prescriptions	ATC: C02A-C, C02DA, C02L, C02DB, C02DD, C02DG, C03A-B, C03D-E, C03X, C07A-D, C07F, C08, C09AA, C09BA, C09BB, C09CA, C09DA, C09DB, C09XA02, C09XA52
Anxiolytic drugs	Prescriptions	ATC: N05BA, N05CF
**Endpoints**		
Fractures	Diagnosis codes	ICD10: S720-729, S321-325, S520-529, S620-628, S420-429
Head injury, major	Diagnosis codes	ICD10: S02, S061-069
Head injury, minor	Diagnosis codes	ICD10: S00, S01, S060, S07

Abbreviations: ICD (International Classification of Diseases system, 10th revision), NCSP (NOMESCO Classification of Surgical Procedures), ATC (Anatomical Therapeutic Chemical system), MI (myocardial infarction), AV block (atrioventricular block), LBBB (left bundle branch block)
