# Supplementary material for: Risk of post-discharge fall-related injuries among adult patients with syncope: A nationwide cohort study
Source: PLoS One. 2018 Nov 21;13(11):e0206936. doi: 10.1371/journal.pone.0206936 (PMC6248940; doi:10.1371/journal.pone.0206936)
Supplement: S2 Table — (PDF) [file pone.0206936.s002.pdf]

S2 Table. Summary of fall-related injures within one year[a]

Syncope (n=125,763)	Total	Age <50 y	Age 50-64 y	Age 65-79 y	Age ≥80 y
Any fall-related injury	8394 (100.0)	1882 (100.0)	1282 (100.0)	2226 (100.0)	3004 (100.0)
Minor head injury[b]	3913 (46.6)	1232 (65.5)	653 (50.9)	924 (41.5)	1104 (36.8)
Wrist or forearm fracture	1491 (17.8)	407 (21.6)	260 (20.3)	399 (17.9)	425 (14.1)
Shoulder or upper arm fracture	822 (9.8)	80 (4.3)	166 (12.9)	278 (12.5)	298 (9.9)
Major head injury[c]	562 (6.7)	137 (7.3)	89 (6.9)	166 (7.5)	170 (5.7)
Hip fracture	1606 (19.1)	26 (1.4)	114 (8.9)	459 (20.6)	1007 (33.5)
**No syncope (n=125,763)**	**Total**	**Age <50 y**	**Age 50-64 y**	**Age 65-79 y**	**Age ≥80 y**
Any fall-related injury	4049 (100.0)	669 (100.0)	477 (100.0)	986 (100.0)	1917 (100.0)
Minor head injury[b]	1383 (34.2)	344 (51.4)	195 (40.9)	291 (29.5)	553 (28.8)
Wrist or forearm fracture	1040 (25.7)	245 (36.6)	169 (35.4)	284 (28.8)	342 (17.8)
Shoulder or upper arm fracture	428 (10.6)	29 (4.3)	63 (13.2)	136 (13.8)	200 (10.4)
Major head injury[c]	182 (4.5)	44 (6.6)	19 (4.0)	41 (4.2)	78 (4.1)
Hip fracture	1016 (25.1)	7 (1.0)	31 (6.5)	234 (23.7)	744 (38.8)

[a]Age refers to age at inclusion

[b]Defined as concussion, superficial contusion, or wound

[c]Defined as skull fracture or traumatic intracranial bleeding
