# Supplementary figures and images for: Risk of post-discharge fall-related injuries among adult patients with syncope: A nationwide cohort study
Source: PLoS One. 2018 Nov 21;13(11):e0206936. doi: 10.1371/journal.pone.0206936 (PMC6248940; doi:10.1371/journal.pone.0206936)

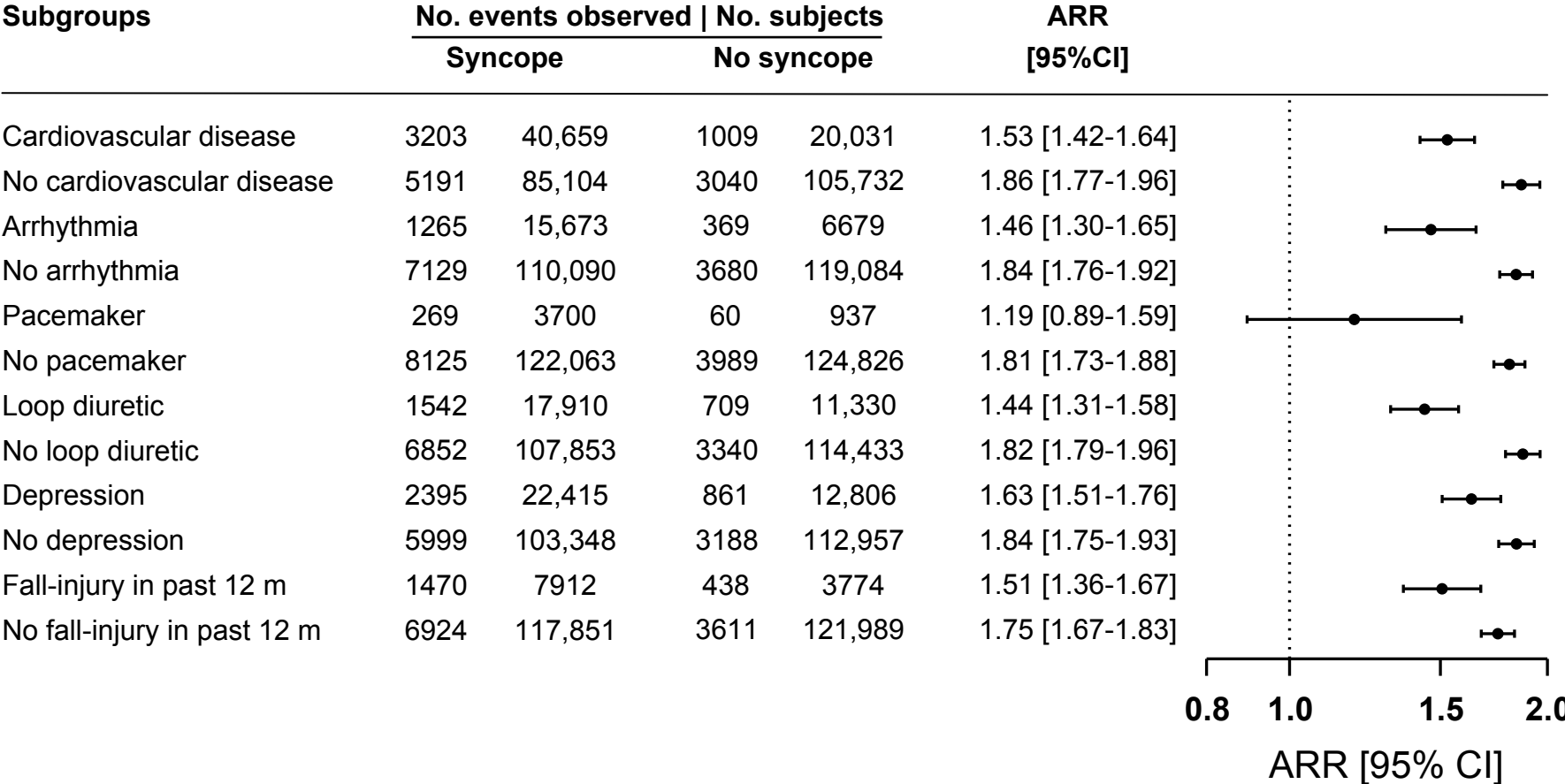

Supplement: S1 Fig — The age- and sex matched group without prior syncope served as reference in all analyses. Multiple absolute risk regression analyses with adjustment for: age, sex, calendar year, socioeconomic status, comorbidities, and pharmacotherapy. ARR indicates absolute risk ratio. (PDF) [file pone.0206936.s003.pdf]

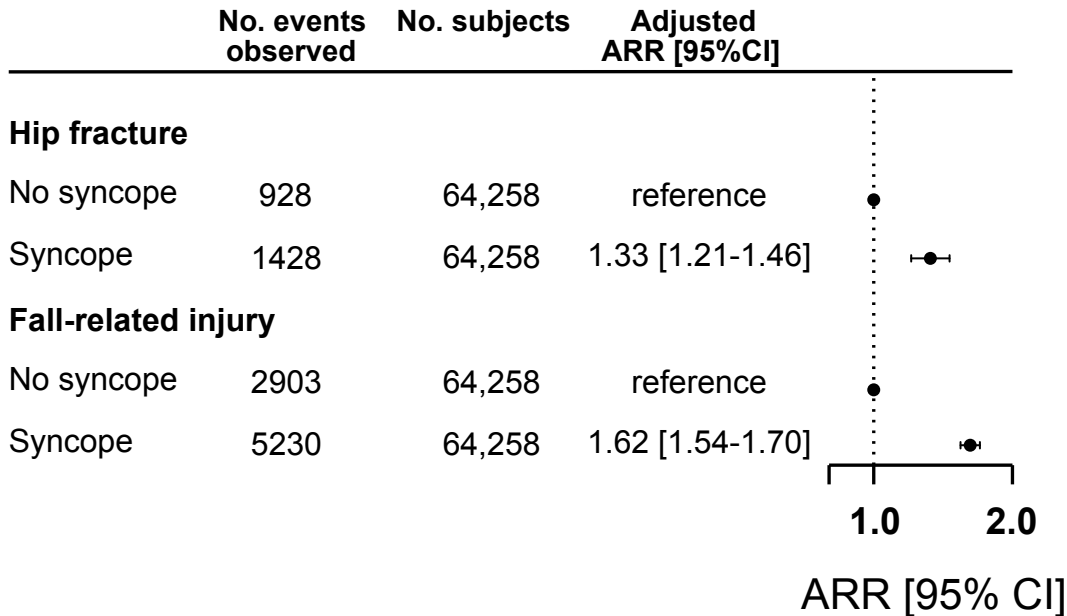

Supplement: S2 Fig — The age- and sex matched group ≥65 years served as reference. Multiple absolute risk regression analyses with adjustment for: age, sex, calendar year, socioeconomic status, ischemic heart disease, arrhythmia, atrioventricular block or left bundle branch block, pacemaker, use of antihypertensive, loop diuretic or anxiolytic drugs, depression, diabetes, cancer, Parkinson disease, and dementia. ARR for total fall-related injury is provided for comparative purpose. ARR indicates absolute risk ratio. (PDF) [file pone.0206936.s004.pdf]
